# Supplementary material for: Heat adaptation of phage T7 under an extended genetic code
Source: Virus Evol. 2021 Dec 1;7(2):veab100. doi: 10.1093/ve/veab100 (PMC8923235; doi:10.1093/ve/veab100)
Supplement: veab100_Supp [file veab100_supp.zip › Sequencing_Analysis.zip!/Sequencing_Analysis/A1/evidence/SNP_11.html]

BRESEQ :: Evidence


| Predicted mutation | | | | | | |
| --- | --- | --- | --- | --- | --- | --- |
| evidence | seq id | position | mutation | annotation | gene | description |
| RA | NC\_001604.1 | 34,982 | C→T | intergenic (–/–) | – / – | –/– |

| Read alignment evidence... | | | | | | | | | | | |
| --- | --- | --- | --- | --- | --- | --- | --- | --- | --- | --- | --- |
|  | seq id | position | | ref | new | freq | score (cons/poly) | reads | annotation | genes | product |
| \* | NC\_001604.1 | 34,982 | 0 | C | T | 88.5% | 6123.3 / inf | 3029 | intergenic (–/–) | *–/–* | –/– |
| Reads supporting (aligned to +/- strand):  **ref** base C (180/165);  **new** base T (1343/1304);  **total** (1538/1491) | | | | | | | | | | | |
| Fisher's exact test for biased strand distribution *p*-value = 6.47e-01 | | | | | | | | | | | |
| Kolmogorov-Smirnov test that lower quality scores support variant *p*-value = 1.98e-02 | | | | | | | | | | | |

Only 100 of 3064 total aligned reads displayed.  

`TTAATATCCCGTTTGAGTATCTAGCCCGTAAGTTCGTAGTGGTAACTCTTATTGGTGTAGACCGAAAGGTCCTTACGATTAATACAGACTATCGCTTTGCTACACGTACTACTATCTCTCTGACAAAGGCTTGGGGTCCAGCCGATGGCTACACGACCATCGAGTTACGTCGAGTAACCTCCACTACCGACCGATTGGTTGACTTTACGGATGGTTCAATCCTCCGCGCGTATGACCTTAACGTCGCTCAGATTCAAACGATGCACGTAGCGGAAGAGGCCCGTGACCTCACTACGGATACTATCGGTGTCAATAACGATGGTCACTTGGA‑TGCTCGTG‑GTCGTCGAATTGTGAACCTAGCGAACGCCGTGGATGACCGCGATGCTGTTCCGTTTGGTCAACTAAAGACCATGAACCAGAACTCATGGCAAGCACGTAATGAAGCCTTACAGTTCCGTAATGAGGCTGAGACTTTCAGAAACCAAGCGGAGGGCTTTAAGAACGAGTCCAGTACCAACGCTACGAACACAAAGCAGTGGCGCGATGAGACCAAGGGTTTCCGAGACGAAGCCAAGCGGTTCAAGAATACGGCTGGTCAATA  >  NC_001604.1/34682‑35282`  

                                                                                                                                                                                                                                                                                                            `|`                                                                                                                                                                                                                                                                                                                
   `cgtgggctcggaaatgtgtataagagccagtCGTAGTGGTAACTCTTATTGGTGTAGACCGAAAGGTCCTTACGATTGATACAGACTATCGCTTTGCTACACGTACTACTATCTCTCTGACAAAGGCTTGGGGTCCAGCCGATGGCTACACGACCATCGAGTTACGTCGAGTAACCTCCACTACCGACCGATTGGTTGACTTTACGGATGGTTCAATCCTCCGCGCGTATGACCTTAACGTCGCTCAGATTCAAACGATGCACGTAGCGGAAGAGGCCCGTGACCTCACTACGTATACTAt                                                                                                                                                                                                                                                                                                             <  1:339495/271‑1 (MQ=255)`  
   `atatCCCGTTTGAGTATCTAGCCCGTAAGTTCGTAGTGGTAACTCTTATTGGTGTAGACCGAAAGGTCCTTACGATTAATACAGACTATCGCTTTGCTACACGTACTACTATCTCTCTGACAAAGGCTTGGGGTCCAGCCGATGGCTACACGACCATCGAGTTACGTCGAGTAACCTCCACTACCGACAGATTGGTTGACTTTACGGATGGTTCAATCCTCCGCGCGTATGACCTTAACGTCGCTCAGATTCAAACGATGCACGTAGCGGAAGAGGCCCGTGACCTCACTACGTATACTAt                                                                                                                                                                                                                                                                                                             <  1:230880/301‑1 (MQ=255)`  
      `tCCCGTTTGAGTATCTAGCCCGTAAGTTCGTAGTGGTAACTCTTATTGGTGTAGACCGAAAGGTCCTTACGATTAATACAGACTATCGCTTTGCTACACGTACTACTATCTCTCTGACAAAGGCTTGGGGTCCAGCCGATGGCTACACGACCATCGAGTTACGTCCAGTAACCTCCACTACCGACCGATTTGTTGACTTTACGGATCGTTCAATCCTCCGCGCGTATGACCTTCACGTCTCTCAGATTCAATCGATGCACGTAGCGTAAGAGGCCCGTGACCTCCCTCCGGATTTTATctg                                                                                                                                                                                                                                                                                                          >  1:301939/1‑299 (MQ=255)`  
      `tCCCGTTTGAGTATCTAGCCCGTAAGTTCGTAGTGGTAACTCTTATTGGTGTAGACCGAAAGGTCCTTACGATTAATACAGACTATCGCTTTGCTACACGTACTACTATCTCTCTGACAAAGGCTGGGGGTCCAGCCGATGGCTACACTACCATCGAGTTACGTCGAGTAACCTCCACTACCGACCGATTGGTTGACTTTACGGATGGTTCAATCCTCCGCGCGTATGACCTTAACGTCGCTCAGATTCAAACGATGCACGTAGCGGAAGAGGCCCGTGACCTCACTACGGATATTATCgg                                                                                                                                                                                                                                                                                                          <  1:221966/301‑1 (MQ=255)`  
       `cccGTTTGAGTATCTAGCCCGTAAGTTCGTAGTGGTAACTCTTATTGGTGTAGACCGAAAGGTCCTTACGATTAATACAGACTATCGCTTTGCTACACGTACTACTATCTCTCTGACAAAGGCTTGGGGTCCAGCCGATGGCTACACGACCATCCAGTTACGTCGAGTAACCTCCACTACCGACCGATTGGTTGACTTTACGGATGGTTCAATCCTCCGCGCGTATGACTTTAACGTCGCTCAGATTCCAACGATGCACGTAGCGGAAGAGGCCCGTGACCTCACTACGGCTATTATCggg                                                                                                                                                                                                                                                                                                         >  1:14269/1‑300 (MQ=255)`  
        `ccGTTTGAGTATCTAGCCCGTAAGTTCGGAGTGGTAACTCTTATTGGTGTAGACCGAAAGGTCATTAAGATTAATACAGACTATCTCTTTGCTACACGTACTACTATTTCTCTGACAAAGGCTTGGGGTCCAGCCGATGGCTACACGACCATCGAGTTACGTCGAGTAACCTCCACTACCGACCGATTGGTTGACTTTACGGATGGTTCAATCCTCCGCGCGTATGACCTTAACGTCGCTCAGATTCAAACGATGCACGTAGCGGAAGAGGCCCGTGACCTCACTACGGATATTATAGgtg                                                                                                                                                                                                                                                                                                        <  1:82218/301‑1 (MQ=255)`  
                 `tATCTAGCCCGTAAGTTCTTAGTGGTAACTCTTAGTGGTGTAGACCGAAAGGTCCATACGATTAATACAGACTATCGCTTTGCTACACGTACGACGAGCACTGTGCCAAAGGCTTGGGGTCCAGCCGATGGCTACACGACCATCGAGTTACGTCGAGTAACCTCCACTACCGACCGATTGGTTGACTTTACGGATGGTTCAATCCTCCGCGCGTATGACCTTAACGTCGCTCAGATTCAAACGATGCACGTAGCGGAAGAGGCCCGTGACCTCACTACGGATATTATCGGTGTCAATAACg                                                                                                                                                                                                                                                                                               <  1:117118/301‑1 (MQ=255)`  
                 `gacagaGCCCGTAAGTTCGTAGTGGTAACTCTTATTGGTGTAGACCGAAAGGTCCTTACGATTAATACAGACTATCGCTTTGCTACACGTACTACTATCTCTCTGACAAAGGCTTGGGGTCCAGCCGATGGCTACACGACCATCGAGTTACGTCGAGTAACCTCCACTACCGACCGATTGGTTGACTTTACGGATGGTTCAATCCTCCGCGCGTATGACCTTAACGTCGCTCAGATTCAAACGATGCACGTAGCGGAAGAGGCCCGTGACCTCACTACGGATATTATCGGTGTCAATAACg                                                                                                                                                                                                                                                                                               <  1:212526/296‑1 (MQ=255)`  
                  `gagatgtgtataagagacaggTGGTAACTCTTATTGGTGTAGACCGAAAGGTCCTTACTATTAATACAGACTATCGCTTTGCTACACGTACTACTATCTCTCTGACAAAGGCTTGGGGTCCAGCCGATGGCTACACGACCATCGAGTTACGTCGAGTAACCTCCACTACCGACCGATTGGTTGACTTTACGGATGGTTCAATCCTCCGCGCGTATGACCTTAACGTCGCTCAGATTCAAACGATGCACGTAGCGGAAGAGGCCCGTGACCTCACTACGGATATTATCGGCGTCAATAACGa                                                                                                                                                                                                                                                                                              <  1:384957/281‑1 (MQ=255)`  
                         `cctgtatttCGTAGTGTTAACTCTTATTGGTGTAGACCGAAAGGTCCTTACGATTAATACAGACTATCGCTTTGCTACACGTACTACTATCTCTCTGACAAAGGCTTGGGGTCCAGCCGATGGCTACACGACCATCGAGTTACGTCGAGTAACCTCCACTACCGACCGATTGGTTGACTTTACGGATGGTTCAATCCTCCGCGCGTATGACCTTAACGTCGCTCAGATTCAAACGATGCACGTAGCGGAAGAGGCCCGTGACCTCACTACGGATATTATCGGTGTCAATAACGATGGTCAc                                                                                                                                                                                                                                                                                       <  1:490794/294‑1 (MQ=255)`  
                              `aGTTCGTAGTGGTAACTCTTATTGGTGTAGACCGAAAGGTCCTTACGATTAATACAGACTATCGCTTTGCTACACGTACTACTATCTCTCTGACAAAGGCTTGGGGTCCAGCCGATGGCTACACGACCATCGAGTTACGTCGAGTAACCTCCACTACCGACAGATTGGTTGACTTTACGGAAGGTTCAATCCTCCGCGCGTATGACCTTAACGTCGCTCCAATTCAAACGATGCACGTAGCGGAAGAGGCCCGTGACATCACTATGGATATTATCGGTGTCAATAACGCTGGTCACTTggt                                                                                                                                                                                                                                                                                  >  1:498440/1‑300 (MQ=255)`  
                                 `tCGTAGTGGTAACTCTTATTGGTGTAGACCGAAAGGGCCTTACGATTAATACAGACTATCGCTTTGCTACACGTACTACTATCTCTCTGACAAAGGCTTGTGGTCCAGCCGATGGCTACACGACCATCGAGTTACGTCGAGTAACCTCCACTACCGACCGATTGGTTTACTTTACGGATGGTTCAATCCTCCGCGCGTATGACCTTAACGTCGCTCAGATTCAAACGATGCACGTAGCGGAAGAGGCCCGTGACCTCACTACGGATATTATCGGTGTCAATAACGATGGTCACTTGGA‑TGc                                                                                                                                                                                                                                                                              <  1:68175/301‑1 (MQ=255)`  
                                  `cgtgatgttagctcgTATTGGTGAGGGCCGAAAGGTCCTTAACATTAATACGGACTATCGCTTTGCTACACGTACTACTATCTCTCTGACGAGGGCTTGGGGTCCAGCCGATGGCTACACGACCATCGAGTTGCGTCGAGTAACCTCCACTACCGACCGATTGGTTGACTTTACGGATGGTTCAATCCTCCGCGCGTATGACCTTAACGTCGCTCAGATTCAAACGATGCACATAGCGGAAGAGGCCCGTGACCTCACTACGGATATTATCGGTGTCAATAACGATGGTCACTTGGA‑TGca                                                                                                                                                                                                                                                                             <  1:151277/290‑2 (MQ=255)`  
                                      `gTGGTAACTCTTATTGGTGTAGACCGAAAGGTCCTTACGATTAATACAGACTATCGCTTTGCTACACGTACTACTATCTCTCTGACAAAGGCTTGGGGTCCAGCCGATGGCTACACGACCATCGAGTTACGTCGAGTAACCTCCACTACCGACCGATTGGTTGACTTTACGGATGGTTCAATCCTCCGCGCGTATGACCTTAACGTCGCTCAGATTCAAGCGATGCACGTAGCGGAAGAGGCACGTGACCTCACTACGGATATTATCGGTGTCAATACCGATGGTCACTTGGA‑TGATCGTg                                                                                                                                                                                                                                                                         >  1:177223/1‑301 (MQ=255)`  
                                      `gTGGTAACTCTTATTGGTGTAGACCGAAAGGTCCTTACGATTAATACAGACTATCGCTTTGCTACACGTACTACTATCTCTCTGACAAAGGCTTGGGGTCCAGCCGATGGCTACACGACCATCGAGTTACGTCGAGTAACCTCCACTACCGACCGATTGGTTGACTTTACGGATGGTTCAATCCTCCGCGCGTATGACCTTAACGTCGCTCAGATTCAAACGATGCACGTAGCGGAAGAGGCCCGTGACCTCACTACGGATATTATCGGTGTCAATAACGATGGTCACTTGGA‑TGCTCGTg                                                                                                                                                                                                                                                                         >  1:45616/1‑301 (MQ=255)`  
                                      `gTGGTAACTCTTATTGGTGTAGACCGAAAGGTCCTTACGATTAATACAGACTATCGCTTTGCTACACGTACTACTAACTCTCTGACAAAGGCTTGGGGTCCAGCCGATGGCTACACGACCATCGAGTTACGTCGAGTAACCTCCACTACCGACCGATTGGTTGACTTTACGGATGGTTCAATCCTCCGCGCGTATGACCTTAACGTCGCTCAGATTCAAACTATGCACGTAGCGGAAGAGGCCCGTTACCTCACTACGGATATTATCGGTGTCCATAACGACTGTCTCttatacccctctt                                                                                                                                                                                                                                                                          >  1:273134/1‑290 (MQ=255)`  
                                       `tGGTAACTCTTATTGGTGTAGACCGAAAGGTCCTTACGATTAATACAGACTATCGCTTTGCTACACGTACTACTATCTCTCTGACAAAGGCTTGGGGTCCAGCCGATGGCTACACGACCATCGAGTTACGTCGAGTAACCTCCACTACCGACCGATTGGTTGACTTTACGGATGGTTCAATCCTCCGCGCGTATGACCTTAACGTCGCTCAGATTCAAACGATGCACGTAGCGGAAGAGGCCCGTGACCTCACTACGTATACTATCGGTGTCAATAACGATGGTCACTTGGA‑TGCTCGTg‑g                                                                                                                                                                                                                                                                       >  1:100242/1‑301 (MQ=255)`  
                                        `ggTAACTCTTATTGGGGTAGACCGAAAGGTCCTTACGATTAATACAGACTATCGCGTTGCTACACGTACTACTATCTCTCTGACAAAGGCTTGGGGTCCAGCCGATGGCTACACGACCATCGAGTTACGTCGAGTAACCTCCACTACCGACCGATTGGTTGACTTTACGGATGGTTCAATCCTCCGCGCGTATGACCTTAACGTCGCTCAGATTCAAACGATGCACGTAGCGGAAGAGGCCCGTGACCTCACTGCGGATATTATCGGTGTCAATAACGATGGTCACTTGGA‑TGCTCGTG‑gt                                                                                                                                                                                                                                                                      <  1:281571/301‑1 (MQ=255)`  
                                             `ctctTATTGGTGTAGACCGAAAGGTCCTTACGCTTAATACAGACTATCGCTTTGCTACACGTACTACTATCTCTCTTACAACGGCTTGTGGTCCAGCCGATGGCTACACGACCATCGAGTTACGTCGCTTAACCTCCACTACCGCCCGATTGGTTGACTTTACGGATGGTTCTATCCTCCGCGCGTGTGCACTTAAAGTCGCTCAGATTCAAACGATTCACGTAGCGGAAGAGACCCGTGCACTCACTACCGATATTTACGTTGTCACTAACGATTGTCACTCGGA‑GGCTCGTgattcttg                                                                                                                                                                                                                                                                  >  1:226360/1‑294 (MQ=255)`  
                                             `ctctTATTGGTGTAGACCGAAAGGTCCTTACGATTAATACAGACTATCGCTTTGCTACACGTACTACTATCTCTCTTTCAAAGGCTTTTTTTCCCGCCGATTTCTACACGACCATCTTTTTACGTCGTTTCACCTCCACTACCGCCCCATTGGTTTACTTTTCGGCTGGTTCTATCCTTCTCTCGTATTTCCTTAACGTCTCTCAGCTTCAATCTCTTCACTTTGCTTTATTGGCCCCTTACCTCTCTACGTATATTATCTGTTTTATTAActtttctccctttgtttctcttcttctttt                                                                                                                                                                                                                                                                   >  1:353560/1‑272 (MQ=255)`  
                                             `ctctTATTGGTGTAGACCGAAAGGTCCTTACGATTAATACAGACTATCGCTTTGCTACACGTACTACTATCTCTCTGACAAAGGCTTGGGGTCCAGCCGATGGCTACACGACCATCGAGTTACGTCGAGTAACCTCCACTACCGACCGATTGGTTGACTTTACGGATGGTTCAATCCTCCGCGCGTATGACCTTAACGTCGCTCAGATTCAAACGATGCACGTAGCGGAAGAGGCTCGTGACCTCACTACGGAGATTATCGGTGTCAATAACGATGGTCACTTGGA‑TGCTCGTG‑Gtcgtcg                                                                                                                                                                                                                                                                 >  1:208647/1‑301 (MQ=255)`  
                                             `ctctTATTGGTGTAGACCGAAAGGTCCTTACGATTAATACAGACTATCGCTTTGCTACACGTACTACTATCTCTCTGACAAAGGCTTGGGGTCCAGCCGATGGCTACACGACCATCGAGTTACGTCGAGTAACCTCCACTACCGACCGATTGGTTGACTTTACGGATGGTTCAATCCTCCGCGCGTATGACCTTAACGTCGCTCAGATTCAAACGATGCACGTAGCGGAAGAGGCCCGTGACCTCACTACGGATATTATCGGTGTCAATAACGATGGTCACTTGgc‑tgctgtctcttatac                                                                                                                                                                                                                                                                  >  1:505640/1‑290 (MQ=255)`  
                                               `ctTATTGGTGTAGACCGAAAGGTCCTTACGATTAATACAGACTATCGCTTTGCTACACGTACTACTATCTCTCTGACAAAGGCTTGGGGTCCAGCCGATGGCTACACGACCATCGAGTTACGTCGAGTAACCTCCACTACCGACCGATTGGTTGACTTTACGGATGGTTCAATCCTCCGCGCGTATGACCTTAACGTCGCTCAGATTCAAACGATGCCCGTAGCGGAAGAGGCCCGTGACCTCACTACGGCTATTCTCTGTTTCAATAACGATGGTCACTTGGA‑TGCTCGTT‑GTCGTCGaa                                                                                                                                                                                                                                                               >  1:344184/1‑301 (MQ=255)`  
                                                `taatgggTGTAGACAGAAAGGTCCGTACAATTAAGACAGATATTCGCTTTTCTACTCTTACTACTATCTCTCTGACAAATGCTTGTGGTCCAGCCGATGGCTATACGACCATCGAGTTTCGTCGAGTAACCTCTACTACCGACCGTTTGGTTGACTTTACGGATTGTTCAATCCTCCGCGCGTATGACCTTAACGTCGCTCAGATTCAAGCGATGCACGTAGCGGAAGAGGCCCGTGACCTCACTACGGATATTAGCGGTGTCAATAACGATGGTCACTTGGA‑TGCTCGTG‑GTCGTCGAAt                                                                                                                                                                                                                                                              <  1:248680/296‑1 (MQ=255)`  
                                                      `gtgtAGACCGAAAGGTCCTTACGATTAATACAGACTATCGCTTTGCTACACGTACTACTATCTCTCTGACAAAGGCTTGGGGTCCAGCCGATGGCTACACGACCATCGAGTTACGTAGAGTAACCTCCACTACCGACCGATTGGTTGACTTTACGGATGGTTCAATCCTCCGCGCGTATGACCTTAACGTCGCTCAGATTCAAACGATGCACGTAGCGGAAGAGGCCCGTGACCTCACTACGGAGATTATCGGTGTCAATAACGATGGTCACTTGGA‑TGCTCGTG‑GTCTTCGAATTGTGaa                                                                                                                                                                                                                                                        >  1:90069/1‑301 (MQ=255)`  
                                                                      `gagatgtgtataagagacagaTCGCTTTGCTACACGTACTACTATCTCTCTGACAAAGGCTTGGGGTCCAGCCGATGGCTACACGACCATCGAGTTACGTCGAGTAACCTCCACTACCGACCGATTGGTTGACTTTACGGATGGTTCAATCCTCCGCGCGTATGACCTTAACGTCGCTCAGATTCAAACGATGCACGTAGCGGAAGAGGCCCGTGACCTCACTACGGATATTATCGGTGTCAATAACGATGGTCACTTGGA‑TGCTCGTG‑GTCGTCGAATTGTGAACCTAGCGAACGCCGTg                                                                                                                                                                                                                                        <  1:147123/281‑1 (MQ=255)`  
                                                                          `gcGATTAATACAGACTATCGCTTTGCTACACGTTCTACTATCTCTCTGACAAAGGCTTGGGGTCCAGCCGATGGCTACACGACCATCGAGTTACGTCGAGTAACCTCCACTACAGACCGATTGGTTGACTTTACGGATGGTTCAATCCTCCGCGCGTATGACATTAACGTCGCTCAGATTCAAACGATGCACGTAGCGGAAGAGGCCCGTGACCTCACTACGGATATTATCGGTGTCAATAACGATGGTCACTTGGA‑TGCTCGTG‑GTCGTCGAATTGTGAACCTAGCGAACGCCGTGGATg                                                                                                                                                                                                                                    <  1:460161/300‑1 (MQ=255)`  
                                                                           `cGATTAATACAGACTATCGCTTTGCTACACGTACTACTATCTCTCTGACAAAGGCTTGGGGTCCAGCCGATGGCTACACGACCATCGAGTTACGTCGAGTAACCTCCACTACCGACCGATTGGTTGACTTTACGGATGGTTCAATCCTCCGCGCGTATGACATTAACGTCGCTCAGATTCAAACGATGCACGTAGCGGATGCGGCCCGTGACCTCACTACGGATATTATCGGTGTCAATAACGTTGGTCACTTGGC‑TGCTCGTG‑TTCGTGGAATTGTGAACCTAGAGAACGCCGTGGATGa                                                                                                                                                                                                                                   >  1:125798/1‑301 (MQ=255)`  
                                                                            `gATTAATCCAGACTATCGCTTTGCTACACGTTCTACTATCTCTCTGACAAAGGCTTGGGGTCCAGCCGATGGCTACACGACCATCGAGTTACGTCGAGTAACCTCCACTACCGACCGATTGGTTAACTTTACGGATGGTTCAATCCTCCGCGCGTATGACCTTAACGTCGCTCAGATTCAAACGATGCACGTAGCGGAAGAGGCCCGTGACCTCACTACGGATATTATCGGTGTCAATAACGATGGTCACTTGGA‑TGCTCGTG‑GTCGTCGAATTGTGAACCTAGCGAACGCCGTGGATGAc                                                                                                                                                                                                                                  <  1:267743/301‑1 (MQ=255)`  
                                                                            `gATTAATACAGACTATCGCTTTGCTACACGTACTACTATCTCTCTGACAAAGGCTTGGGGTCCAGCCGATGGCTACACGTCCATCGAGTTACGTCGAGTAACCTCCACTACCGACCGATTGGTTAACTTTACGGATGGATCAATCCTCCGCGCGTATGACCTTAACGTTGCTCAGATTCAAACGATGCACGTAGCGGAAGAGACCCGTGACCTCACTACGTATATTATCGATGTCAATAACGCTGGCCACTTGGA‑TGCTCGTG‑TTCGCCGCATTGTGAACCTAGCGACCGCCGTGGATAAc                                                                                                                                                                                                                                  >  1:495130/1‑301 (MQ=255)`  
                                                                            `gATTAATACAGACTATCGCTTTGCTACACGTACTACTATCTCTCTGACAAAGGCTTGGGGTCCAGCCGATGGCTACACGACCATCGAGTTACGTCGAGTAACCTCCACTACCGACCGATTGGTTGACTTTACGGATGGTTCAATCATCCGCGCGTATGACCTTAACGTCGCTAAGATTCAAACGATGCACGTAGCGGAAGAGGCCCGTGACCTCACTACGGATATTATCGGTGTCAATAACGATGGTCACTTGGA‑TGCTCGTG‑GTCGTCGAACtgtctcttatacccatctccgagcccac                                                                                                                                                                                                                                  >  1:415147/1‑276 (MQ=255)`  
                                                                                 `aTACAGACTATCGCTTTGCTACACGTACTACTATCTCTCTGACAAAGGCTTGGGGTCCAGCCGATGGCTACACGACCATCGAGTTACGTCGAGTAACCTCCACTACCGACCGATTGGTTGACTTTACGGATGGTTCAATCCTCCGCGCGTATGACCTTAACGTCGCTCAGATTCAAACGATGCACGTAGCGGAAGAGGCCCGTGACCTCACTACTGATATTATTGGTGTCAATAACGATGGTCACTTGGA‑TGCTCGTG‑GTCGTCGAATTGTGAACCTAGCGAACACCTTGGATTACctcgt                                                                                                                                                                                                                             >  1:333061/1‑300 (MQ=255)`  
                                                                                       `aCTATCGCTTTGCTACACGTACTACTATCTCTCTGACAAAGGCTTGGGGTCCAGCCGATGGCTACACGACCATCGAGTTACGTCGAGTAACCTCCACTACCTACCGATTGGTTGACTTTACGGATGGTTCAATCCTCCGCGCGTATGACCTTAACGTCGCTCAGATTCAAACGATGCACGTAGCGGAAGAGGCCCGTGACCTCACTACTGATATTATCGGTGTCAATAACGATGGTCACTTGGA‑TGCTCGTG‑GTCGTCGAATTGTGAACCTAGCGAACGCCGTGGATGACCGCGATGCTGt                                                                                                                                                                                                                       <  1:9559/301‑1 (MQ=255)`  
                                                                                         `tATCGCTTTGCTACACGTACTACTATCTCTCTGACAAAGGCTTGGGGTCCAGCCGATGGCTACACGACCATCGAGTTACGTCGAGTAACCTCCACTACCGACCGATTGGTTGACTTTACGGATGGTTCAATCCTCCGCGCGTATGACCTTAACGTCGCTCAGACTCAAACGATGCACGTAGCGGAAGAGGCCCGTGACCTCACTACGGATATTATCGGTGTCAATAACGATGGTCACTTGGA‑TGCTCGTG‑GTCGTCGAATTGTGAACCTAGCGAACGCCGTGGATGACCGCGATGCTGTTc                                                                                                                                                                                                                     <  1:237655/301‑1 (MQ=255)`  
                                                                                          `aTCGCTTTGCTACACGTACTACTATCTCTCTGACAAAGGCTTGGGGTCCAGCCGATGGCTACACGACCATCGAGTTACGTCGAGTAACCTCCACTACCGACCGATTGGTTGACTTTACGGATGGTTCAATCCTCCGCGCGTATGACCTTAACGTCGCTCCGAGTAAAACGATGCACGTAGCGGAAGAGGCCCGTGACCGCACTACGGATATTATAGGTGTCAATAACGATGGTCACTTGGA‑TGCTCGTG‑GTCGTCGAATTGTGAACCTAGCGAACGCCGAGGGCTACCGCGATGCTGTGcc                                                                                                                                                                                                                    >  1:320463/1‑301 (MQ=255)`  
                                                                                             `ataagagacagcGTACTACTATCTCTCTGACAAAGGATTGGGGTCCAGCCGATGGCTACACGACCATCGAGTGACGTCGAGTAACCTCCACTACCGACCGATTGGTTGACTTTACGGATGGTTCAATCCTACGCGCGTATGACCTTAACGTCGCTCAGATTCAAACGATGCACGTAGCGGAAGAGGCCCGTGACCTCACTACGGATATTATCGGTGTCAATAACGATGGTCACTTGGA‑TGCTCGTG‑GTCGTCGAATTGTGAACCTAGCGAACGCCGTGGATGACCGCGATGCTGTTCCGtt                                                                                                                                                                                                                 <  1:55391/290‑1 (MQ=255)`  
                                                                                              `cTTTGCTACACGTACTACTATCTCTCTGACAAAGGCTTGGGGTCCAGCCGATGGCTACACGACCATCGAGTTACGTCGAGTAACCTCCACTACCGACCGATTGGTTAACTTTACGGATGGTTCAATCCTCCGCGCGTATGACCTTAACGTCGCTCAGATTCAAACGATGCACGTAGCGGAAGAGGCCCGTGACCTCACTACGGATATTATCGGTGTCAATAACGATGGTCACTTGGA‑TGCTCGTG‑GTCGTCGAATTGTGAACCTAGCGAACGCCGTGGATGACCGCGATGCTGTTCCGttt                                                                                                                                                                                                                <  1:390051/301‑1 (MQ=255)`  
                                                                                                `ttGCTACACGTACTACTATCTCTCTGACAAAGGCTTGGGGTCCAGCCGATGGCTACACGACCATCGAGTTACGTCGAGTAACCTCCACTACCGACCGATTGGTTGACTTTACGGATGGTTCAATCCTCCGCGCGTATGACCTTAACGTCGCTCAGATTCAAACGATGCACGTAGCGGAAGAGGCCCGTGACCTCACTACGGATATTATCGGTGTCAATAACGATGGTCACTTGGA‑TGCTCGTG‑TTCGTCGAATTGTGAACCTAGCGAACGCCGTGGATgctgtctcttatacacatctccg                                                                                                                                                                                                              >  1:217750/1‑279 (MQ=255)`  
                                                                                                `ttGCTACACGTACTACTATCTCTCTGACAAAGGCTTGGGGTCCAGCCGATGGCTACACGACCATCGAGTTACGTCGAGTAACCTCCACTACCGACCGATTGGTTGACTTTACGGATGGTTCAATCCTCCGCGCGTATGACCTTAACGTCGCTCAGATTCAAACGATGCACGTAGCGGAAGAGGCCCGTGACCTCACTACGGATATTATCGGTGTCAATAACGATGGTCACTTGGA‑TGCTCGTG‑GTCGTCGAATTGTGAACCTAGCGAACGCCGTGGATGACCGCGATGCTGTTCCGTTTgg                                                                                                                                                                                                              >  1:182602/1‑301 (MQ=255)`  
                                                                                                 `tGCTACACGTACTACTATCTCTCTTACAAAGGCTTGGGTTCCAGCCGATGGCTACACGA‑CATCGAGTTACGTCGAGTAACCTCCACTACCGACCGATTGGTTGACTTTACGGATGGTTCAATCCTCCGCGCGTATGACCTTAACGTCGCTCAGATTCAAACGATGCACGTAGCGGAAGAGGCCCGTGACCTCACTACGTATACTATCGGTGTCAATAACGATGGTCACTTGGA‑TGCTCGTG‑GTCGTCGAATTGTGAACCTAGCGAACGCCGTGGATGACCGCGATGCTGTTCCGTTTGGTc                                                                                                                                                                                                            <  1:324137/301‑1 (MQ=255)`  
                                                                                                 `ataagagacagctactaTCTCTCTGACAAAGGCTTGGGGTCCAGCCGATGGCTACACGACAATCGAGTTACGTCGAGTAACCTCCACTACCGACCGATTGGTTGACTTTACGGATGGTTCAATCCTCCGCGCGTATGACCTTAACGTCGCTCAGATTCAAACGATGCACGTAGCGGAAGAGGCCCGTGACCTCACTACGGATATTATCGGTGTCAATAACGATGGTCACTTGGA‑TGCTCGTG‑GTCGTCGAATTGTGAACCTAGCGAACGCCGTGGATGACCGCGATGCTGTTCCGTTTGGt                                                                                                                                                                                                             <  1:434741/290‑1 (MQ=255)`  
                                                                                                     `acccGTACTACTATCTCTCTGACAAAGGCTTGGGTTCCAGCCGATGGCTACACGACCATCGAGTTACGTCGAGTAACCTCCACTACCGACCGATTGGTTGACTTTACGGATGGTTCAATCCTCCGCGCGTATGACCTTAACGTCGCTCAGATTCAAACGATGCACGTAGCGGAAGAGGCCCGTGACCTCACTACGGATATTATCGGTGTCAATAACGATGGTAACTTGGA‑TGCTCGTG‑GTCGTCGAATTGTGAACCTAGCGAACACCGTGGATGACCGCGATGCTGTTCCGTTTGGTCAAc                                                                                                                                                                                                         <  1:419911/298‑1 (MQ=255)`  
                                                                                                       `acGTACTACTATCTCTCTGACAAAGGCTTGGGGTCCAGCCGATGGCTACACGACCATCGAGTTACGTCGAGTAACCTCCACTACCGACCGCTTGGTTGACTTTACGGATGGTTCAATCCTCCGCGCGTATGACCTTAACGTCGCTCAGATTCAAACGATGCACGTAGCGGAAGAGGCCCGTGACCTCACTACGGATATTATCGGTGTCAATAACGATGGTCACTTGGA‑TGCTCGTG‑GTCGTCGAATTGTTAACCTAGCGAACGCCGTGGATGACCGCGATGCGGTTcctgtctcttataca                                                                                                                                                                                                       >  1:3154/1‑288 (MQ=255)`  
                                                                                                        `cGTACTACTATCTCTCTGACAAAGGCTTGGGGTCCAGCCGATGGCTACACGACCATCGAGTTACGTCGAGTAACCTCCACTACCGACCGATTGGTTGACTTTACGGTTGGTTCAATCCTCCGCGCGTATGACCTTAACGTCGATCAGATTCAAACGATGCAGGTAGCGGAAGAGGCCCGTGACCTAAAGACGGATATTATCAGTTTAAATAACGAAGGTAACTTGGA‑TGC‑GGTGTGTCGACGAATTGTGAACGCAGCGAACGACGTGGATGACCCTGATGCTGTTCCGTTGGGGCAACTaa                                                                                                                                                                                                      >  1:51613/1‑301 (MQ=255)`  
                                                                                                         `ggactactATCTCTCTGACAAAGGCTTGGGGTCCAGCCGATGGCTACACGACCATCGAGTTACGTCGAGTAACCTCCACTATCGACCGATTGATTAACTTTACGGATGGTTCAATCCTCTTCGCGTAAGACCTTAACGTCGCTCAGATTCCAACGATGCACGTCGCGGAATAGGCCCGTGACCTCACTACGGGAATTATCCGTGTTACTCACGTTCGTCACTTTGATTACCCGT‑‑GTCGGCGAATTGTTCACCTTTCGACCTCCGTTGTGTACCGCCATGCTTTTCCTTTTGGTTAACTaat                                                                                                                                                                                                     >  1:109924/3‑300 (MQ=255)`  
                                                                                                         `gTACTACTATCTCTCTGACAAAGGCTTGGGGTCCAGCCGATGGCTACACGACCATCGAGTTACGTCGAGTAACCTCCACTACCGACCGATTGGTTGACTTTACGGATGGTTCAATCCTCCGCGCGTATGACCTTAACGTCGCTCAGATTCAAACGATGCACGTAGCGGAAGAGGCCCGTGACCTCACTACGGATATTATCGGTGTCAATAACGATGGTCACTTGGA‑TGCTCGTG‑GTCGTCGAATTGTGAACCTAGCGAACGCCGTGGATGACCGCGATGCTGTTCCGTTTGGTCAACTaaa                                                                                                                                                                                                     >  1:327751/1‑301 (MQ=255)`  
                                                                                                            `ctactaTCTCTCTGACAAAGGCTTGGGGTCCAGCCGATGGCTACACGACCATCGAGTTACGTCGAGTAACCTACACTACCGACCGATTGGTTGACTTTACGGATGGTTCAATCCTCCGCGCGTATGACCTTAACGTCGCTCAGATTCAAACGATGCACGTAGCGGAAGAGGCCAGTGACCTCACTACGGATATTATCGGTGTCAATAACGATGGTCACTTGGA‑TGCTCGTG‑GTCTTCGAATTGTGAACCTAGCGAACGCCGTGGATGACCGCGATCCTGTTCCGTTTGGTCAACTAACGAc                                                                                                                                                                                                  >  1:465343/1‑301 (MQ=255)`  
                                                                                                            `ctactaTCTCCCTGACAAAGGCTTGGGGTCCAGCCGATTGCTACACGACCATCGAGTTACGTCGACTAACCTCCACTACCGACCGATTGGTTGACTTTACGGATGGTTCAATCCTCCGCGCGTATGACCTTAACGTCGCTCAGATTCAAACGATGCACGTAGCGGAAGAGGCCCGTGACCTCACTACGGATATTAGCGGTGTCAATAACGATGGTCACTTGGA‑TGCTCGTG‑GTCGTCGAATTGTGAACCTAGCGAACGCCGTGGATGACCGCGATGCTGTTCCGTTTGGTCAACTAAAGAc                                                                                                                                                                                                  <  1:483181/301‑1 (MQ=255)`  
                                                                                                            `ctactaTCGTGCTGACAAAGGCTTGGGGTACAGCCGATGGCTACACGACCATCGAGTTACGTCGAGTAACCGCCACTACCGACCGATTGGTTGACTTTACGGATGGTTCAATCCTCCGAGAGTATGACCTTAACGTCGCTCAGATTCAAACGATGCACGTAGCGGAAGAGGCACGTGACCTCACTACGGATATTATCGGTGTCAATAACGATGGTCACTTGGA‑TGCTCGTG‑GTCGTCGAATTGTGAACCTAGCGAACGCCGTAGATGACCGCGATGCTGTTCCGTTTGGTCAACTAAAGAc                                                                                                                                                                                                  <  1:374939/301‑1 (MQ=255)`  
                                                                                                            `agactaTCTCTCTGACAAAGGCTTGGGGTCCAGCCGATGGCTACACGACCATCGAGTTACGTCGAGTAACCTCCACTACCGACCGATTGGTTGACTTTACGGATGGTTCAATCCTCCGCGCGTATGACCTTAACGTCGCTCATATTCAAACGATGCACGTAGCGGAAGAGGCCCGTGACCTCACTACGTATACTATCGGTGTCAATAACGATGGTCACTTGGA‑TGCTCGTG‑GTCGTCGAATTGTGAACCTAGCGAACGCCGTGGATGACCGCGATGCTGTTCCGTTTGGTCAACTAAAGAc                                                                                                                                                                                                  <  1:439120/299‑1 (MQ=255)`  
                                                                                                              `actaTCTCTCTGACAAAGGCTTGGGGTCCAGCCGATGGCTACACGACCATCGAGTTACGTCGAGTAACCTCCACTACCGACCGATTGGTTGACTTTACGGATGGTTCAATCCTCCGCGCGTATGACCTTAACGTCGCTCAGATTCAAACGATGCACGCAGCGGAAGAGGCCCGTGACCTCACTACGGATATTATCGGTGTCAATAACGATGGTCACTTGGA‑TGCTCGTG‑GTCGTCGAATTGTGAACCTAGCGAACGCCGTGGATGACCGCGATGCTGTTCCGTTTGGTCAACTAAAGAccc                                                                                                                                                                                                >  1:406613/1‑300 (MQ=255)`  
                                                                                                                    `agatgtgtataagagacaggTCCAGCCGATGGCTACACGACCATCGAGTTACGTCGAGTAACCTCCACTACCGACCGATTGGTTGACTTTACGGATGGTTCAATCCTCCGCGCGTATGACCTTAACGTCGCTCAGATTCAAACGATGCACGTAGCGGAAGAGGCCCGTGACCTCACTACGTATACTATCGGTGTCAATAACGATGGTCACTTGGA‑TGCTCGTG‑GTCGTCGAATTGTGAACCTAGCGAACGCCGTGGATGACCGCGATGCTGTTCCGTTTGGTCAACTAAAGACCATGGAcc                                                                                                                                                                                          <  1:26299/283‑1 (MQ=255)`  
                                                                                                                         `gaaaaaGGCTTGGGGTCCAGCCGATGGCTACACGACCATAGAGTTACGTCGAGTAACCTCCACTACCGACCGATTGGTTAACTTTACGGATGGTTCAATCATCCGCGCGTATGACCTTAACGTCGCTCAGATTCAAACGATGCACGTAGCGGAAGAGGCCCGTGACCTCACTACGGATATTATCGGTGTCAATAACGATGGTCACTTGGA‑TGCTCGTG‑GTCGTCGAATTGTGAACCTAGCGAACGCCGTGGATGACCGCGATGCTGTTCCGTTTGGTCAACTAAAGACCATGGACCAGAAc                                                                                                                                                                                     <  1:501773/298‑1 (MQ=255)`  
                                                                                                                         `gACAAAGGCTTGGGGTCCAGCCGATGGCTACACGACCATCGAGTTTCGTAGAGTAACCTCCACTACCGACCGATTGGTTGACTTTACGGATGGTTCAATCCTCCGCGCGTATGACCTTAACGTCGCTCAGATTCAAACGATGCACGTAGCGGAAGAGGCCCGTGACCTCACTACGGATATTATCGGTGTCAATAACGATGGTCACTTGGA‑TGCTCGTG‑GTCGTCGAATTGTGACCCTAGCGAACGCCGTGGATGACCGCGCTGCTGTTCCGTTTGGTCAACTAAAGACCATGGACCAGAAc                                                                                                                                                                                     >  1:277814/1‑301 (MQ=255)`  
                                                                                                                              `aGGCTGTGGGTCCAGCCTATGGCTACACGACCATCGAGTTACGTCGAGTAACCTCCACTACCGACCGATTGGTTAACTTTACGGATGGTTCAATCCTCCGCGCGTATGACCTTAACGTCGCTCAGATTCAAACGATGCACGTAGCGGAAGAGGCCCGTGACCTCACTACGGATATTATCGGTGTCAATAACGATGGTCACTTGGA‑TGCTCGTG‑GTCGTCGAATTGTGAACCTAGCGAACGCCGTGGATGACCGCGATGCTGTTCCGTTTGGTCAACTAAAGACCATGGACCAGAACTCATg                                                                                                                                                                                <  1:48665/301‑1 (MQ=255)`  
                                                                                                                               `ggCTTGGGGTCCAGCCGATGGCTACACGACCATCGAGTTACGTCGAGTAACCTCCACTACCGACCGATTGGTTGACTTTACAGATGGTTCAATACTCCGCGCGTATGACCTTAACGTCGCTCAGATTCAAACGATGCACGTAGCGGAAGAGGCCCGTGACCTCACTACGGATATTATCGGTGTCAATGACGATGGTCACTTGGA‑TGCTCGTG‑GTCGTCGAATTGTGAACCTAGCGAACGGCGGGGATGACGGCGCTGATGTTCCGTTTGGTCAACTAAAGACCCGGAACCAAAACGCATgg                                                                                                                                                                               >  1:285144/1‑301 (MQ=255)`  
                                                                                                                                             `atgtgtagaagagacagattgAGTTACGTCGAGTAACCTCCACTACCGACCGATTGGTTGACTTTACGGATGGTTCAATCCTCCGCGCGTATGACCTTAACGTCGCTCAGATTCAAACGATGCACGTAGCGGAAGAGGCCCGTGACCTCACTACGGATATTATCGGTGTCAATAACGATGGTCACTTGGA‑TGCTCGTG‑GTCGTCGAATTGTGAACCTAGCGAACGCCGTGGATGACCGCGATGCTGTTCCGTTTGGTCAACTAAAGACCATGGACCAGAACTCATGGCAAGCACGTAATGa                                                                                                                                                                 <  1:104902/281‑1 (MQ=255)`  
                                                                                                                                               `gATGGCTACACGACCATCGAGTTACGTCGAGTAACCTCCACTACCGACCGATTGGTTGACTTTACGGATGGTTCAATCCTCCGCGCGTATGACCTTAACGTCGCTCAGATTCAAACGATGCACGTAGCGGAAGAGGCCCGTGACCTCACTACGTATACTATCGGTGTCAATAACGATGGTCACTTGGA‑TGCTCGTG‑GTCGTCGAATTGTGAACCTAGCGAACGCCGTGGATGACCGCGATGCTGTTCCGTTTGGTCAACTAAAGACCATGGACCAGAACTCATGGCAAGCACGTAATGAAg                                                                                                                                                               <  1:477389/301‑1 (MQ=255)`  
                                                                                                                                                 `tGGCTACACGACCATCGAGTTACGTCGAGTAACCTCCACTACCGACCGATTGGTTGACTTTACGGATGGTTCAATCCTCCGCGCGTATGACCTTAACGTCGCTCAGATTCAAACGATGCACGTAGCGGAAGAGGCCCGTGACCTCACTACGGATATTATCGGTGTCAATAACGATGGTCACTTGGA‑TGCTCGTG‑GTCGTCGAATTGTGAACCTAGCGAACGCCGTGGATGACCGCGATGCTGTTCCGTTTGGTCAACTAAAGACCATGGACCAGAACTCATGGCAAGCACGTAAttcactt                                                                                                                                                             >  1:64504/1‑295 (MQ=255)`  
                                                                                                                                                     `aacacGACCATCTAGTTACGTCGAGTAACCTCCACTACCGACCGATTGGTTGACTTTACGGATGGTTCAATCCTCCGCGCGTATGACCTTAACGTCGCTCAGATTCAAACGATGCACGTAGCGGAAGAGGCCCGTGACCTCACTACGGATATTATCGGTGTCAATAACGATTGTCACTTGGA‑TGCTCGTG‑GTCGTCGAATTGTGAACCTAGCGAACGCCGTGGATGACCGCGATGCTGTTCCGTTTGGTCAACTAAAGACCATGGACCAGAACTCATGGCAAGCACGTAATGAAGACTTAc                                                                                                                                                         <  1:137396/300‑1 (MQ=255)`  
                                                                                                                                                       `aaagACCATCGAGTTACGGCGAGTAAGCTCCACTACCGACCGTTTGGTTGACTTTACGGATGGTTCAATCCTCCGCGCGTATGAACTTAACGTCGCTCAGATTCAAACTATGCACGTAGCGGAAGAGGCCCGTGACCTCACTACGGATATTATCGGTGTCAATAACGATGGTCACTTGGA‑TGCTCGTG‑GTCGTCGAATTGTGAACCTAGCGAACGCCGTGGATGACCGCGATGCTGTTCCGTTTGGTCAACTAAAGACCATGGACCAGAACTCATGGCAAGCACGTAATGAAGCCTTACAg                                                                                                                                                       <  1:348749/298‑1 (MQ=255)`  
                                                                                                                                                          `gACCATCGAGTTACGTCGAGTAACCTCCACTACCGACCGATTGGTTGACTTTACGGATGGTTCAATCCTCCGCGCGTATGACCTTAACGTCGCTCAGATTCAAACGATGCACGTAGCGGAAGAGGCCCGTGACCTCACTACGGATATTATCGGTGTCAATAACGATGGTCACTTGGA‑TGCTCGTG‑GTCGTCGAATTGTGAACCTAGCGAACGCCGTGGATGACCGCGATGCTGTTCCGTTTGGTCAACTAAAGACCATGGACCAGAACTCATGGCAAGCACGTAATGAAGCCTTACAGTTc                                                                                                                                                    <  1:161582/301‑1 (MQ=255)`  
                                                                                                                                                               `tCGAGTTACGTCGAGTAACCTCCACTACCGACCGATTGGTTGACTTTACGGATGGTTCAATCCTCCGCGCGTATGACCTTAACGTCGCTCAGATTCAAACGATGCACGTAGCGGAAGAGGCCCGTGACCTCACTACGGATATTATCGGTGTCAATAACGATGGTCACTTTGA‑TGCTCGTG‑GTCCTCGAATTGTGAACCTAGCGAACGCCGTGGATGACCGCGATGCTGTTCCGTTTGGTCAACTAAAGACCATGGACCAGAACTCATGGCAAGCACGTAATGAAGCCTTACAGTTCCGTaa                                                                                                                                               <  1:428585/301‑1 (MQ=255)`  
                                                                                                                                                               `tCGAGTTACGTCGAGTAACCTCCACTACCGACCGATTGGTTGACTTTACGGATGGTTCAATCCTACGCGCGTATGACCTTAACGTCGCTCAGATTCAAACGATGCACGTAGCGGAAGAGGCCCGTGACCTCACTACGGATATTATCGGTGTCAATAACGATGGTCACTTGGA‑TGCTCGTG‑GTCGTCGAATTGTGAACCTAGCGAACGCCGTGGATGACCGAGATGCTGTTCCGTTTGGTCAACTAAAGACCATGGACCAGAACTCATGGCAAGCACGTAATGAAGCCTTACAGTTCCGTaa                                                                                                                                               <  1:444959/301‑1 (MQ=255)`  
                                                                                                                                                               `tCGAGTTACGTCGAGTAACCTCCACTACCGACCGATTGGTTAACGTTACGGATGGTTCAATCCTCCGCGCGTATGACCTTAACGTCGCTCAGATTAAAACGATGCACGGAGCGGAAGAGGCCCGTGACCTCACTACGGATATTATCGGTGTCAATAACGATGGTCACTTGGA‑TGCTCGTG‑GTCGTCGAATTGTGAACCTAGCGAACGCCGTGGATGACCGCGATGCTGTTCCGGTTGGTCAACTAAAGACCATGGACCAGAACTCATGGCAAGCACGTAATGAAGCCTTACAGTTCCGTaa                                                                                                                                               <  1:41127/301‑1 (MQ=255)`  
                                                                                                                                                                `cGAGTTACGTCGAGTAACCTACACTACCGACCGATTGGTTGACTTTACGGATGGTTCAATCCTCCGCGCGTATGACCTTAACGTCGCTCAGATTCAAACGATGCACGTAGCGGAAGAGGCCCGTGACCTCACTACGTATACTATCGGTGTCAATAACGATGGTCACTTGGA‑TGCTCGTG‑GTCGTCGAATTGTGAACCTAGCGAACGCCGTGGATGACCGCGATGCTGTTCCGTTTGGTCAACTAAAGACCATGGACCAGAACTCATGGCAAGCACGTAATGAAGCCTTACAGTTCCATAAt                                                                                                                                              <  1:171207/301‑1 (MQ=255)`  
                                                                                                                                                                     `tACGTCGAGGAACCTCCACGACCGACCGAGTGGGTGACTTTACGGATGGTTCAATCCTCCGCGGGTATGACCTTAACGTCGCTCAGATTCAAACGATGCACGTAGCGGAAGAGGCCCGTGAGCTCACTACGGATATTATCGGTGTCAATAACGATGGTCACTTGGA‑TGCTCGTG‑GTCGTCGAATTGTGAACCTAGCGAACGCCGTGGATGACCGCGATGCTGTTCCGTTTGGTCAACTAAAGACCATGGACCAGAACTCATGGCAAGCACGTAATGAAGCCTTACAGTTCCGTAATGAGGc                                                                                                                                         <  1:425112/301‑1 (MQ=255)`  
                                                                                                                                                                      `aCGTCGAGTAACCTCCACTACCGACCGATTGGTTAACTTTACGGATGGTTCAATCCTCCGCGCGTATGACCTTAACGTCGCTCAGATTCAAACGATGCACGTAGCGGAAGAGGCCCGTGACCTCACTACGGATATTATCGGTGTCAATAACGATGGTCACTTGGA‑TGCTAGTG‑GTCGTCGAATTGTGAACCTAGCGAACGCCGTGGATGACCGCGATGCTGTTCCGTTTGGTCAACTAAAGACCATGGACCAGAACTCATGGCAAGCACGTAATGAAGCCTTCCAGTTCCGTaactgtctc                                                                                                                                        >  1:120855/1‑294 (MQ=255)`  
                                                                                                                                                                                 `ccTCCACTACCGACCGATTGGTTGACTTTACGGATGGTTCAATCATCCGCGCGTATGACCTTAACGTCGCTCAGATTCAAACGATGCACGTAGCGGAAGAGGCCCGTGACCTCACTACGTATACTATCGGTGTAAATAACGATGGTCACTTGGA‑TGCTCGTG‑GTCGTCGAATTGTGAACCTAGCGAACGCCGTGGATAACCGCGATGCTGTTCCGTTTGGTCAACTAAAGACCATGGACCAGAACTCATGGCAAGCACGTAATGAAGACTTACAGTTCCGTAATGCGGCTGAGACTTTCAg                                                                                                                             >  1:378384/1‑301 (MQ=255)`  
                                                                                                                                                                                 `ccTCCACTACCGACCGATTGGTTAACTTTACGGATGGTTCAATCCTCCGCGCGTATGACCTTAACGTCGCTCAGATTCAAACGATGCACGTAGCGGAAGAGGCCCGTGACCTCACTACGGATATTATCGGTGTCAATAACGATGGTCACTTGGA‑TGCTCGTG‑GTCGTCGAATTGTGAACCTAGCGAACGCCGTGGATGACCGCGATGCTGTTCCGTTTGGTCAACTAAAGACCATGGACCAGAACTCATGGCAAGCACGTAATGAAGCCTTAcctgtctcttatacacatctccgagccca                                                                                                                             >  1:508269/1‑273 (MQ=255)`  
                                                                                                                                                                                     `cACTACCGACCGATTGGTTGACTTTACGGATGGTTCAATCCTCCGCGCGTATGACCTTAACGTCGCTCAGATTCAAACGATGCACGTAGCGGAAGAGGCCCGTGACCTCACTACGGATATTATCGGTGTCAATAACGATGGTCACTTGGA‑TGCTCGTG‑GTCGTTGAATTGTGAACCTAGCGAACGCCGTGGATGACCGCGATGCTGTTCCGTTTGGTCAACTAAAGACCATGGACCAGAACTCATGGCAAGCACGTAATGAAGCCTTACAGTTCCGTAATGAGGCTGAGACTTTCAGAAAc                                                                                                                         <  1:241565/301‑1 (MQ=255)`  
                                                                                                                                                                                             `aacagttGGTTGACTTTACGGATGGTTCAATCCTCCGCGCGTATGACCTTAACGTCGCTCAGATTCAAACGATGCACGTAGCGGAAGAGGCCCGTGACCTCACTACGGATATTATCGGTGTCAATAACGATGGTCACTTGGA‑TGCTCGTG‑GTCGTCGAATTGTGAACCTAGCGAACGCCGTGGATGACCGCGATGCTGTTCCGTTTGGTCAACTAAAGACTATGGACCAGAACTCATGGCAAGCACGTAATGAAGCCTTACAGTTCCGTAATGAGGCTGAGACTTTCAGAAACCAAGCGGa                                                                                                                 <  1:76559/296‑1 (MQ=255)`  
                                                                                                                                                                                              `ccgaTTGGTTGACTTTACGGATGGTTCAATCCTCCGCGCGTATGACCTTAACGTCGCTCAGATTCAAACGATGCACGTAGCGGAAGAGGCCCGTGACCTCACTACGGATATTATCGGTGTCAATAACGATGGTCACTTGGA‑TGCTCGTG‑GTCGTCGAATTGTGAACCTAGCGAACGCCGTGGATGACCGCGATGCTGTTCCGTTTGGTCAACTAAAGACCATGGACCAGAACTCATGGCAAGCACGTAATGAAGCCTTACAGTTCCGTAATTAGGCTGAGACTTTCAGAAACCAAGCGGAg                                                                                                                >  1:364780/1‑301 (MQ=255)`  
                                                                                                                                                                                                       `tGACTTTACGGATGGTTCAATCCTCCGCGCGTATGACCTTAACGTCGCTCAGATTCAAACGATGCACGTAGCGGAAGAGGCCCGTGACCTCACTACGGATATTATCGGTGTCAATAACGATGGTCACTTGGA‑TGCTCGTG‑GTCGTCGAATTGTGAACCTAGCGAACTCCGTGTATGACCGCGATGCTGTTCCGTTTGGTCAACTAAAGACCATGGACCAGAACTCATGGCAAGCACGTAATGAAGCCTTACAGTTCCGTAATGAGGCTGAGACTTTCAGAAACCAAGCGGAGGGCTTTAAg                                                                                                       <  1:165163/301‑1 (MQ=255)`  
                                                                                                                                                                                                       `ggtataagagacaggTTCAATCCTCCGCGCGTATGACCTTAACGTCGCTCAGATTCAAACGATGCACGTAGCGGAAGAGGCCCGTGACCTCACTACGGATATTATCGGTGTCAATAACGATGGTCACTTGGA‑TGCTCGTG‑GTAGTCGAATTGTGAACCTAGCGAACGCCGTGGATGACCGCGATGCTGTTCCGTTTGGTCAACTAAAGACCATGGACCAGAACTCATGGCAAGCACGTAATGAAGCCTTACAGTTCCGTAATGAGGCTGAGACTTTCAGAAACCAAGCGGAGGGCTTTAAg                                                                                                       <  1:156071/288‑1 (MQ=255)`  
                                                                                                                                                                                                         `tcTTTACGGATGGTTCACTCCTCCGCCCGGATGACCTTAACGTCGCTCAGATTCAAACGATGCACGTAGCGGAAGAGGCCCGTGACCTTACTACGGATATTATCGGTGTCAATAACGATTGTCACTTGGA‑TGATCGTG‑GTCGTCGAATTGTGAACCTAGCGAACGCCGGGGATGACCGCGATGCTGTTCCGTTTGGTCAACTAAAGACCATGGACCAGAACTCATGGCAAGCACGTAATGAAGCCTTACAGTTCCGTAATGAGGCTGAGACTTTCAGAAACCAAGCGGAGGGCTTTAAGaa                                                                                                     <  1:298908/300‑1 (MQ=255)`  
                                                                                                                                                                                                         `gcTTTACGGATGGTTCAATCCTCCGCGCGTATGACCTTAACGTCGATCAGATTCAAACGATGCACGTAGCGGAAGAGGCCCGTGACCTCACTACGGATATTATCGGTGTCAATAACGATGGTCACTTGGA‑TGCTCGTG‑GTCGTCGAATTGTGAACCTAGCGAACGCCGTGGATGACCGCGATGCTGTTCCGTTTGGTCAACTAAAGACCATGGACCAGATCTCATGGCAAGCACGTAATGAAGCCTTACAGTTCCGTAATGAGGCTGAGACTTTCAGAAACCAAGCGGAGGGCTTTAAGaa                                                                                                     <  1:358848/300‑1 (MQ=255)`  
                                                                                                                                                                                                                     `gTTCAATCCTCCGCGCGTATGACCTTAACGTCGCTCAGATTCAAACGATGCACGTAGCGGAAGAGGCCCGTGACCTCACTACGGATATTATCGGTGTCAATAACGATGGTCACTTGGA‑TGCTCGTG‑GTCGTCGAATTGTGAACCTAGCGAACGCCGTGGATGACCGCGATGCTGTTCCGTTTGGTCAACTAAAGACCATGGACCAGAACTCATGGCAAGCACGTAATGAAGCCTTACAGTTCCGTAATGAGGCTGAGACTTTCAGAAACCAAGCGGAGGGCTTTAAGAACGAGTCCAGTAc                                                                                         >  1:395209/1‑301 (MQ=255)`  
                                                                                                                                                                                                                     `gTTCAATCCTACGCGCGTATGACCTTAACGTCGCTCAGATTCAAACGATGCACGTAGCGGAAGAGGCCCGTGACCTCACTACGGATATTATCGGTGTCAATAACGATGGTCACTTGGA‑TGCTCGTG‑GTCGTCGAATTGTGAACCTAGCGAACGCCGTGGATGACCGCGATGCTGTTCCGTTTGGTCAACTAAAGACCATGGACCAGAACTCATGGCAAGCACGTAATGAAGCCTTACAGTTCCGTAATGAGGCTGAGACTTTCAGAAACCAAGCGGAGGGCTTTAAGAAcctgtctcttat                                                                                         >  1:306863/1‑290 (MQ=255)`  
                                                                                                                                                                                                                       `tCAATCCTCCGCGCGTATGACCTTAACGTCGCTAAGATTCAAACGATGCACGTAGCGGAAGAGGCCCGTGACCTCACTACGGATATTATCGATGTCAATAACGATGGTCACTTGGA‑TGCTCGTG‑GTCGTCGAATTGTGAACCTAGCGAACGCCGTGGATGACCGCGATGCTGTTCCGTTTGGTCAACTAAAGACCATGGACCAGAACTCATGGCAAGCACGTAATGAAGCCTTACAGTTCCGTAATGAGGCTGAGACTTTCAGAAACCAAGCGGAGGGCTTTAAGAACGAGTCCAGTACCa                                                                                       >  1:200881/1‑301 (MQ=255)`  
                                                                                                                                                                                                                           `ccagccGCGCGTATGACCTTAACGTCGCTCAGATTCAAACGATGCACGTAGCGGAAGAGGCCCGTGACCTCACTACGGATATTATCGGTGTCAATAATGATTGTCACTTGGA‑TGCTCGTG‑GTCGTCGAATTGTGAACCTAGCGAACGCCGTGGATGACCGCGATGCTGTTCCGTTTGGTCAACTAAAGACCATGGACCAGAACTCATGGCAAGCACGTAATGAAGCCTTACAGTTCCGTAATGAGGCTGAGACTTTCAGAAACCAAGCGGAGGGCTTTAAGAACGAGTCCAGTACCAACGc                                                                                   <  1:450349/297‑1 (MQ=255)`  
                                                                                                                                                                                                                                      `tATGACCTTAACGTCGCTCAGATTCAAACGATGCACGTAGCGGAAGAGGCCCGTGACCTCACTACGGATATTATCGGTGTCAATAACGATGGTCACTTGGA‑TGCTCGTG‑GTCGTCGAATTGTGAACCTAGCGAACGCCGTGGATGACCGCGATGCTGTTCCGTTTGGTCAACTAAAGACCATGGACCAGAACTCATGGCAAGCACGTAATGAAGCCTTACAGTTCCGTAATGAGGCTGAGACTTTCAGAAACCAAGCGGAGGGCTTTAAGAACGAGTCCAGTACCAACGCtgtctcttata                                                                        >  1:402207/1‑291 (MQ=255)`  
                                                                                                                                                                                                                                            `cTTAACTTGCCTCAGATTCAAACGAGGCACGTAGCGGAAGAGGCCCGTGACCTCACGACGAATATTATCGGTGTCAATAACGATGGTCACTTGGA‑TGCTCGTG‑GTCGTCGAATTGTGAACCGATCGAACGCCGTGGATGACCGCGATGCTGTTCCGTTTGGGCAACTAAAGACCATGGACCAGAACTCAGGGCAAGCACGTAATGAAGCCTTACAGTTCCGTAATGAGGCTGAGACTTTCAGAAACCAAGCGGAGGGCTTTAAGAACGAGTCCAGTACCAACGCTACGAACACAAAGCAGt                                                                  <  1:60160/301‑1 (MQ=255)`  
                                                                                                                                                                                                                                              `tAACGTCGCTCAGATTCAAACGATACACGTAGCGGAAGAGGCCCGTGACCTCACTACGGATATTATCGGTGTCAATAACGATGGTCACTTGGA‑TGCTCGTG‑GTCGTCGAATTGTGAACCTAGCGAACGCCGTGGATGACCGCGATGCTGTTCCGTTTGGTCAACTAAAGACCATGGACCAGAACTCATGGCAAGCACGTAATGAAGCCTTACAGTTCCGTAATGAGGCTGAGACTTTCAGAAACCAAGCGGAGGGCTTTAAGAACGAGTCCAGTACCAACGCTACGAACACAAAGCAGTgg                                                                <  1:28935/301‑1 (MQ=255)`  
                                                                                                                                                                                                                                                     `gCTCAGATTCAAACGATGCACGTAGCGGAAGAGGCCCGTGACCTCACTACGGATATTATCGGTGTCAATAACGATGGTCACTTGGA‑TGCTCGTG‑GTCGTCTAATTGTGAACCTAGCGAACGCCGTGGATGACCGCGATGCTGTTCAGTTTGGTCAACTAAAGACCATGGACCAGAACTCATGGCAAGCACGTACTGAAGCCTTACAGTTCCGTAATGAGGATGAGACTTTCAGAAACCAAGCGGAGGGCTTTAAGAACGAGTCCAGTACCAACGCTACGAACACAAAGCAGtctgtctctt                                                         >  1:473675/1‑292 (MQ=255)`  
                                                                                                                                                                                                                                                          `gATTCAAACGATGCACGTAGCGGAAGAGGCCCGTGACCTCACTACGTATACTATCGGTGTCAATAACTATGGTCACTTGGA‑TGCTCGTG‑TTCGTCGAATTGTGAACCTAGCGCACGCCGTGGATGACCGCGATGCTGTTCCGTTTGGTCAACTAAAGACCATGGACCAGAACTCATGGCAAGCACGTAATGAAGCCTTACAGTTCCGTAATGAGTCTGAGACTTTCAGAAACCACGCGGAGGGCTTTAAGACCGAGTCCATTACCAACGCTACTAACACAAAGCAGTGGCGCGATGAGAcc                                                    >  1:253820/1‑301 (MQ=255)`  
                                                                                                                                                                                                                                                               `aaaCGATGCACGTAGCGGAAGAGGCCCGTGACCTCACTACGGATATTATCGGTGTCAATAACGATGGTCACTTGGA‑TGCTCGTG‑GTCGTCGAATTGTGAACCTAGCGAACGCCGGGGATTACCGCGATGCTGTTCCGTTTGGTCAACTAAAGACCATGGACCAGAACTCATGGCAAGCACGTAATGAAGCCTTACAGTTCCGTAATGAGGCTGAGACTTTCAGAAACCAAGCGGAGGGCTTTAAGAACGAGTCCAGTACCAACGCTACGAACACAAAGCAGTGGCGCGATGAGACCAAggg                                               <  1:205010/301‑1 (MQ=255)`  
                                                                                                                                                                                                                                                                `aaCGATGCACGTAGCGGAAGAGGCCCGTGACCTCACTACGGATATTATCGGTGTCAATAACGATGGTCACTTGGA‑TGCTCGTG‑GTCGTCGAATTGTGAACCTAGCGAACGCCGTGGATGACCGCGATGCTGTTCCGTTTGGTCAACTAAAGACCATGGACCAGAACTCATGGCAAGCACGTAATGAAGCCTTACAGTTCCGTAATGAGGCTGAGACTTTCAGAAACCAAGCGGAGGGCTTTAAGAACGAGTCCAGTACCAACGCTACGAACACAAAGCAGTGGCGCGATGAGACCAAGGGt                                              >  1:131803/1‑301 (MQ=255)`  
                                                                                                                                                                                                                                                                              `gggAAGAGGCCCGTGACCAAACTACGGATATTATCGGTGTAAATAACGATGGTAACTTGGA‑TGCTCGTG‑GTAGAAGAATTGTGAACATAGAGAACGCCGTGGAAGACCGCGATGCTGTTCAGTTTGGTAAAATAAAGACCATGGAACAGAACTCATGGCAAGCACGTAATGAAGCCTTACAGTTCCGTAATGAGGCTGAGAATTTCAGAAACAAAGAGGAGGGATTTAAGAACGAGTCCAGTACCAACGCTACGAACACAAAGCAGTGGCGCGATGAGACCAAGGGTTTCCGAGACGAAGc                                <  1:369261/300‑1 (MQ=255)`  
                                                                                                                                                                                                                                                                                             `acatCACTACGGATATTGTCGGTGTCAATAAGGATGGTCACTTGGA‑TGCTCGTG‑GTCGTCGAATTGGGAACCTAGCGAACGCCGTGGATGACCGCGATGCTGTTCCGTTTGGTCAACTAAAGACCATGGACCAGAACTCATGGCAAGCACGTAATGAAGCCTTACAGTTCCGTAATGAGGCTGAGACTTTCAGAAACCAAGCGGAGGGCTTTAAGAACGAGTCCAGTACCAACGCTACGAACACAAAGCAGTGGCGCGATGAGACCAAGGGTTTCCGAGACGAAGCCGAGCGGTTCAAGaa                 <  1:173716/298‑1 (MQ=255)`  
                                                                                                                                                                                                                                                                                             `aCCTCACTACGGATATTATCGGTGTCAATAACGATGGTCACTTGGA‑TGCTAGTG‑GTCGTCGAATTGTGAACCTAGCGAACGCCGTGGATGACCGCGATGCTGTTCCGTTTGGTCAACTAAAGACCATGGACCAGAACTCATGGCAAGCACGTAATGAAGCCTTACAGTTCCGTAATGAGGCTGAGACTTTCAGAAACCAAGCGGAGGGCTTTAAGAACGAGTCCAGTACCAACGCTACGAACACAAAGCAGTGGCGCGATGAGACCAAGGGTTTCCGAGACGAAGCCAAGCGGTTCAAGaa                 <  1:189258/301‑1 (MQ=255)`  
                                                                                                                                                                                                                                                                                              `ccTCACTACGGATATTATCGGTGTCAATAACGATGGTCACTTGGA‑TGCTCGTG‑GTCGTCGAATTGTGAACCTAGCGAACGCCGTGGATGACCGCGATGCTGTTCCGTTTGGTCAACTAAAGACCATGGACCAGAACTCATGGCAAGCACGTAATGAAGCCTTACAGTTCCGTAATGAGGCTGAGACTTTCAGAAACCAAGCGGAGGGCTTTAAGAACGAGTCCAGTACCAACGCTACGAACACAAAGCAGTGGCGCGCTGAGACCAAGGGTTTCCGAGACGAAGCCAAGCGGTTCAAGaac                >  1:469313/1‑300 (MQ=255)`  
                                                                                                                                                                                                                                                                                                 `cACTACGTATACTATCGGTGTCAATAACGATGGTCACTTGGA‑TGCTCGTG‑GTCGACGAATTGTGAACCTAGCGAACGCCGTGGATGACCGCGATGCTGTTCCGTTTGGTCAACTAAAGACCATGGACCAGAACTCATGGCAAGCACGTAATGAAGCCTTACAGTTCCGTACTGAGGCTGAGACTTTCAGAAACCAAGCGGCGGGCTTTAAGAACGAGTCCAGTACCAACGCGACGAACACAAAGCTGTTGCGCGATGAGACCACGCGCTTTCGCGACGAAACCAAGCGGTTCAAGAATACg             >  1:293733/1‑301 (MQ=255)`  
                                                                                                                                                                                                                                                                                                 `cACTACGGATATTATCGGTGTCAATAACGATGGTCACTTGGA‑TGCTCGTG‑GTCGTCGAATTGTGAACCTAGCGAACGCCGTGGATGACCGCGATGCTGTTCCGTTTGGTCAACTAAAGACCATGGACCAGAACTCATGGCAAGCACGTAATGAAGCCTTACAGTTCCGTAATGAGGCTGAGACTTTCAGCAACCAAGCGGAGGGCTTTAAGCACGAGTACAGTACCAACGCTACGAACACAAAGCAGTGGCGCGATGAGACCAAGGGTTTCCGAGACGCAGCCAAGCGATTCAAGAATACg             >  1:245715/1‑301 (MQ=255)`  
                                                                                                                                                                                                                                                                                                   `gacagtaTAGTATCGGTGTAAATAACGATGGTCACTTGGA‑GGCGCGTG‑GTCGTCTAATTGTGAACCTAGCGAACGCCGTGGATGACCGCGATGCTGTTCCGTTTGGTCAACTAAAGACCATGGACCAGAACTCATGGCAAGCACGTAATGAAGCCTTACAGTTCCGTAATGAGGCTGAGACTTTCAGAAACCAAGCGGAGGGCTTTAAGAACGAGTCCAGTACCAACGCTACGAACACAAAGCAGTGGCGCGATGAGACCAAGGGTTTCCGAGACGAAGCCGAGCGGTTCAAGAATACGGc           <  1:86193/295‑1 (MQ=255)`  
                                                                                                                                                                                                                                                                                                   `caaCGGATATTATCAGGGTCAAGAACGAGTGTCACTGGGA‑TGCTCGTG‑GTCGGCGAAGTGTGAACCTAGCGAACGCCGGGGATGAACGCGATGCTGTTCCGTTGGGTCAACGAAAGACCATAGACCATAACTCATGGCAAGCACGTAATGAAGCCTGACAGTTCCGTAATGAGGCTGAGACGTTCAGAAACCAAGGGGAGGGCTTTAAGAACGAGTCCAGTACCAACTCTACGAACACAAAGCAGTGGCGCGATGAGACCAAGGGTTTCCGAGACGAAGCCGAGCGGTTCAAGAATATGGc           <  1:113697/299‑1 (MQ=255)`  
                                                                                                                                                                                                                                                                                                   `cTACGTATACTATCGGTGTCAATAACGATGGTCACTTGGA‑TGCTCGTG‑GTCGTCGAATTGTGAACCTAGCGAACGCCGTGGATGACCGCGATGCTGTTCCGTTTGGTCAACTAAAGACCATGGACCAGAACTCATGGCAAGCACGTAATGAAGCCTTATAGTTCCGTAATGAGGCTGAGACTTTCAGAAACCAAGCGGAGGGCTTTAAGAACGAGTCCAGTACCAACGCTACGAACACAAAGCAGTGGCGCGATGAGACCAAGGGTTTCCTAGACGAAGCCGAGCggctgtctcttataca           >  1:257694/1‑287 (MQ=255)`  
                                                                                                                                                                                                                                                                                                   `cTACGTATACTATCGGTGTCAATAACGATGGTCACTTGGA‑TGCTCGTG‑GTCGTCGAATTGTGAACCTAGCGAACGCCGTGGATGACCGCGATGCTGTTCCGTTTGGTCAACTAAAGACCATGGACCAGAACTCATGGCAAGCACGTAATGAAGCCTTACAGTTCCGTAATGAGGCTGAGACTTTCAGAAACCAAGCGGAGGGCTTTAAGAACGAGTCCAGTACCAACGCTACGAACACCAAGCAGTGGCGCGATGAGACCAAGGGTTCCCGAGACGAAGCCAAGCGGTTCAAGAATATGGc           >  1:194870/1‑301 (MQ=255)`  
                                                                                                                                                                                                                                                                                                       `ggATATTATCGGTGTCAATAACGATGGTCACTTGGA‑TGCTAGTG‑GTCGTCGAATTGTGAACCTAGCGAACGCCGTGGATGACCGCGATGCTGTTCCGTTTGGTCAACTAAGGACCATGGACAAGAACTAATGGCAAGCACGTAATGAAGCCTTACAGTTCCGTAATGAGGCTGAGACTTTCAGAAACCAAGCGGAGGGCTTTAAGAACGAGTCCAGTACCAACGCTACGAACACAAAGCAGTCGCACGCTGAGACCAAGGGTTTCCGTGACGAAGCCGAGCGGttacagcaatgtatcgtc       >  1:72351/1‑285 (MQ=255)`  
                                                                                                                                                                                                                                                                                                        `gATATTATCGGTGTCAATAACGATGGTCACTTGGA‑TGCTCGTG‑GTCGTTGAATTGTGAACCTAGCGAACGCCGTGGATGACCGCGATGCTGTTCCGTTTGGTCAACTAAAGACCATGGACCAGAACTCATGGCAAGCACGTAATGAAGCCTTACAGTTCCGTAATGAGGCTGAGACTTTAAGAAACCAAGCGGAGGGCTTTAAGAACGAGTCCAGTACCAACGCTACGAACACAAAGCAGTGGCGCGATGAGACCAAGGGTTTCCGAGACGAAGCCGGGCGGTTCAATAATACGGcctgtc      >  1:310833/1‑296 (MQ=255)`  

                                                                                                                                                                                                                                                                                                            `|`                                                                                                                                                                                                                                                                                                                
`TTAATATCCCGTTTGAGTATCTAGCCCGTAAGTTCGTAGTGGTAACTCTTATTGGTGTAGACCGAAAGGTCCTTACGATTAATACAGACTATCGCTTTGCTACACGTACTACTATCTCTCTGACAAAGGCTTGGGGTCCAGCCGATGGCTACACGACCATCGAGTTACGTCGAGTAACCTCCACTACCGACCGATTGGTTGACTTTACGGATGGTTCAATCCTCCGCGCGTATGACCTTAACGTCGCTCAGATTCAAACGATGCACGTAGCGGAAGAGGCCCGTGACCTCACTACGGATACTATCGGTGTCAATAACGATGGTCACTTGGA‑TGCTCGTG‑GTCGTCGAATTGTGAACCTAGCGAACGCCGTGGATGACCGCGATGCTGTTCCGTTTGGTCAACTAAAGACCATGAACCAGAACTCATGGCAAGCACGTAATGAAGCCTTACAGTTCCGTAATGAGGCTGAGACTTTCAGAAACCAAGCGGAGGGCTTTAAGAACGAGTCCAGTACCAACGCTACGAACACAAAGCAGTGGCGCGATGAGACCAAGGGTTTCCGAGACGAAGCCAAGCGGTTCAAGAATACGGCTGGTCAATA  >  NC_001604.1/34682‑35282`

  

Alignment Legend

Aligned base mismatch/match (shaded by quality score): `ATCG/ATCG` `< 3 ≤` `ATCG/ATCG` `< 9 ≤` `ATCG/ATCG` `< 19 ≤` `ATCG/ATCG` `< 34 ≤` `ATCG/ATCG` `< 38 ≤` `ATCG/ATCG`

Unaligned base: `atcg`    Masked matching base: `atcg`    Alignment gap: `‑`    Deleted base: `‑`
